# Atrophic Cardiac Remodeling Induced by Taurine Deficiency in Wistar Rats

**DOI:** 10.1371/journal.pone.0041439

**Published:** 2012-07-23

**Authors:** Mariele Castilho Pansani, Paula S. Azevedo, Bruna Paola Murino Rafacho, Marcos F. Minicucci, Fernanda Chiuso-Minicucci, Sofia Gonçalves Zorzella-Pezavento, Julio Sergio Marchini, Gilberto João Padovan, Ana Angelica Henrique Fernandes, Beatriz B. Matsubara, Luiz S. Matsubara, Leonardo A. M. Zornoff, Sergio A. R. Paiva

**Affiliations:** 1 Internal Medicine Department, Faculdade de Medicina de Botucatu, Universidade Estadual Paulista (UNESP), Botucatu, São Paulo, Brazil; 2 Department of Microbiology and Immunology, Instituto de Biociências de Botucatu, Universidade Estadual Paulista (UNESP), Botucatu, São Paulo, Brazil; 3 Chemistry and Biochemistry Department, Instituto de Biociências de Botucatu, Universidade Estadual Paulista (UNESP), Botucatu, São Paulo, Brazil; 4 Internal Medicine Department, Faculdade de Medicina de Ribeirão Preto, Universidade de São Paulo (FMRP – USP), Ribeirão Preto, São Paulo, Brazil; The Ohio State Unversity, United States of America

## Abstract

**Introduction:**

Micronutrient deficiency is observed in heart failure patients. Taurine, for example, represents 50% of total free amino acids in the heart, and *in vivo* studies have linked taurine deficiency with cardiomyopathy.

**Methods:**

Thirty-four male Wistar rats (body weight = 100 g) were weighed and randomly assigned to one of two groups: Control (C) or taurine-deficient (T (-)). Beta-alanine at a concentration of 3% was added to the animals’ water to induce taurine deficiency in the T (-) group. On day 30, the rats were individually submitted to echocardiography; morphometrical and histopathological evaluation and metalloproteinase activity, oxidative stress and inflammation evaluation were performed. Tissue samples were collected to determine the taurine concentration in the heart.

**Results:**

Taurine deficiency led to decreases in: ventricular wall thickness, left ventricle dry weight, myocyte sectional area, left ventricle posterior wall thickness and ventricular geometry. With regard to heart function, the velocity of the A wave, the ratio between the E and A wave, the ejection fraction, fractional shortening and cardiac output values were decreased in T (-) rats, suggesting abnormal diastolic and systolic function. Increased fibrosis, inflammation and increased activation of metalloproteinases were not observed. Oxidative stress was increased in deficient animals.

**Conclusions:**

These data suggest that taurine deficiency promotes structural and functional cardiac alterations with unique characteristics.

## Introduction

Patients with heart failure (HF) may have different nutritional needs than those with a normal physiological state [1]. There is evidence that patients with HF are deficient in many micronutrients that play important roles in maintaining calcium homeostasis, controlling oxidative stress and regulating energy and protein metabolism [2]. Among these nutrients, taurine is very important. It is involved in biological processes such as bile salt formation, reduction of the levels of pro-inflammatory cytokines in various organs, insulin activity modulation, anti-hypertension, anti-atherogenic action, hepatoprotection and neurotransmission [3], [4], [5], [6], [7], [8].

Taurine accounts for 50% of total free amino acids in the heart [9]. Allard et al. reported that taurine deficiency contributes to HF in cats and dogs [1]. There are many factors involved in cardiac remodeling and progression of HF, including oxidative stress and inflammation [1], [10], [11], [12]. Taurine is described as a nutrient with the following functions in the heart: osmoregulation, indirect regulator of oxidative stress, anti-inflammatory action, stabilizing membranes through direct interactions with phospholipids, maintenance of normal contractile function, modulation of cellular calcium levels, modulator of protein kinases and phosphatases, inhibiting apoptosis, [8], [13].

Therefore, it is possible that taurine modulates morphological and functional cardiac variables. However, the physiological role of this amino acid in the heart is not fully understood. The objective of this study was to assess the role of taurine deficiency in normal rat hearts.

**Table 1:** Echocardiographic morphological data.

	Cn = 17	T(-)n = 17	P
**LVDD(mm)**	6.76 (6.69–7.34)	6.90 (6.76–7.36)	0.654
**LVDD/BW(mm/kg)**	26.4±2.45	26.4±2.17	0.962
**LVWT (mm)**	1.37±0.11	1.24±0.16*	**0.013**
**LVWT/LVDD**	0.20±0.02	0.17±0.02*	**0.016**
**LA (mm)**	4.0±0.61	3.83±0.39	0.356
**AD (mm)**	3.16±0.30	3.13±0.18	0.755
**LA/AD**	1.26±0.17	1.22±0.15	0.489
**LA/BW (mm/kg)**	15.2±2.38	14.4±2.31	0.298
**LVM (g)**	0.34 (0.33–0.43)	0.36 (0.33–0.43)	0.654
**LVMI (g/kg)**	1.94±0.49	1.87±0.32	0.708

Group C: control animals, Group T (-): taurine-deficient animals; LVDD: left ventricular end-diastolic diameter; BW: body weight; LVWT: left ventricular posterior wall thickness; LA: left atrium diameter; AD: aortic diameter; LVM: left ventricle mass; LVMI: left ventricular mass index. Data are expressed as means ± standard deviations or medians (quartile 1– quartile 3). Bold values indicate statistical significance (P≤0.05).

## Materials and Methods

Animals and treatment: Male Wistar rats, 21 days old and weighting 100 g, were used in the study; the animals were housed and cared for in accordance with the National Institute of Health’s Guide for the Care and Use of Laboratory Animals. The experimental protocol was approved by the Animal Ethics Committee of the Botucatu School of Medicine, UNESP, São Paulo, Brazil. The animals were randomly allocated into two groups: the control group (C; n = 17) and the taurine-deficient group (T (-); n = 17). The animals were housed in individual cages; their feeding was monitored daily, and water was administered *ad libitum*. In group T (-), 3% β-alanine (diluted in water), a well known antagonist of taurine transport, was administered [14], [15]. The consumption of water or solution was measured every other day. The animals were weighed weekly. After 30 days, the animals underwent an echocardiographic study. Then, euthanasia was performed via a large dose of pentobarbital, and biological materials were collected for biochemical and morphometric evaluation.

Doppler-echocardiography: After the observation period, all animals were weighed and evaluated by a transthoracic echocardiographic exam [16]. The exams were performed using a commercially available echocardiographic machine (Philips model TDI 5500) equipped with a 12 MHz phased array transducer. Imaging was performed using a 60° sector angle and a 3 cm imaging depth. Left ventricle (LV) end-diastolic dimension (LVDD) and posterior wall thickness (LVWT) were measured at a maximal diastolic dimension, and the end-systolic dimension (LVSD) was taken at the maximal anterior motion of the posterior wall. The left atrium (LA) was measured at its maximal diameter, and the aorta was measured at the end of diastole. The LV systolic function was assessed by calculating the ejection fraction [(LVDD^3^– LVSD^3^)/LVDD^3^], fractional shortening index [(LVDD – LVSD)/LVDD] x 100, cardiac output (CO) (LVDD^3^−LVSD^3^) x heart rate), flow velocity through the aorta (VAO), and cardiac index (CI) (CO divided by body weight). The velocities of transmitral diastolic flow (E and A velocities) were obtained from the apical four-chamber view. The E/A ratio, the isovolumetric relaxation time (IRT), and the isovolumetric relaxation time normalized to heart rate (IRT/RR0.5) were used as indices of LV diastolic function.

**Table 2:** Echocardiographic functional data.

	Group Cn = 17	Group T(-)n = 17	P
**LVSD (mm)**	2.91±0.57	3.42±0.53*	**0.011**
**LVSD/BW (mm/kg)**	12.1 (9.62–13.00)	12.3 (11.10–14.20)*	**0.046**
**HR (bpm)**	340.15±30.13	304.22±25.91*	**<0.001**
**CO (mL/min)**	73.48±9.07	64.72±7.81*	**0.005**
**% FS**	58.012±6.98	51.70±4.82*	**0.004**
**EF**	0.92±0.03	0.88±0.03*	**0.004**
**E wave (cm/s)**	84.70±12.73	83.62±9.34	0.781
**A wave (cm/s)**	64.0 (52.07–75.15)	52.0 (45.75–56.00)*	**0.008**
**E/A**	1.31±0.22	1.62±0.26*	**0.001**
**IRT (ms)**	16.50 (15.0–19.00)	17.0 (15.5–18.25)	0.876
**IRTc (ms)**	42.5±8.8	38.0±5.5	0.084

Group C: control animals, Group T (-): taurine-deficient animals; LVSD: left ventricular systolic diameter; BW: body weight; HR: heart rate; CO: cardiac output; % FS: fractional shortening; EF: ejection fraction; E/A: relationship between the E and A waves; IRT: isovolumetric relaxation time; IRTc: isovolumetric relaxation time normalized by the HR. Data are expressed as means ± standard deviations or medians (quartile 1 - quartile 3). Bold values indicate statistical significance (P≤0.05).

Collection of biological material: Hearts were collected from the experimental animals. The right and left ventricles (including the interventricular septum) were dissected, separated and weighed. The water content and mass of the tissues was calculated by measuring the weight of the fragment after dissection, called the wet weight (WW), and the weight of the same fragment after it was dried for 48 hours in an oven at 65°C, called the dry weight (DW). Thus, the equation [(WW-DW)/WW] × 100 provides information on the water content in the tissue. The LV dry weight was determined by subtracting the water content from the original tissue weight.

Histopathologic study: A morphometric analysis of the myocardium was performed as described previously [17]. Transverse sections of the LV were fixed in 10% buffered formalin and embedded in paraffin, and stained with hematoxylin and eosin (HE) or the collagen-specific stain *Picrosirius* red (Sirius red F3BA in aqueous saturated picric acid). The measurements were obtained from digital images (40× magnification) that were collected with a video camera attached to a Leica microscope; the images were analyzed using Image-Pro Plus 3.0 software (Media Cybernetics; Silver Spring, MD). The myocyte cross-sectional area (CSA) was measured with a digital pad, and the selected cells were transversely cut so that the nucleus was in the center of the myocyte [18]. The interstitial collagen volume fraction was determined for the entire cardiac section that was stained with *Picrosirius* red by analyzing digital images that were captured under polarized light (20× magnification). Perivascular collagen was excluded from this analysis [18].

Determination of concentrations of taurine in the myocardium: Tissue concentrations of taurine were measured by high-performance liquid chromatography, using a Schimadzu ® LC10AD and a Shimadzu RF535 fluorescence detector. Phase A was a 25 mmol/L sodium phosphate solution, pH 6.9, containing 20 mL/L methanol, 20 ml/L acetonitrile, and 20 ml/L tetrahydrofuran, and phase B was a solution of 65% chromatographic-grade methanol, as described by Löser et al. (1988) e Sussman (1988) [19], [20]. The flow of solvent used was 0.8 ml/minute. An adsorbosphere OPA HR C18 column was used for chromatographic separation. For analysis, 10 mL of sample was used, and 200 mL of MeOH was added. This solution was centrifuged, and the supernatant was transferred to another tube, followed by MeOH evaporation. The solution was resuspended in 100 mL of 0.05 N HCl and then stirred and filtered through a 25 µm membrane; it was then injected into the equipment [21].

Metalloproteinase-2 and -9 activity: Metalloproteinase-2 and -9 activity was determined as previously reported by Tyagi et al [22]. Briefly, samples for analysis were prepared by dilution in an extraction sample buffer consisting of 50 mM Tris, pH 7.4, 0.2 M NaCl, 0.1% Triton-X and 10 mM CaCl_2_. The samples were then diluted in application sample buffer consisting of 0.5 M Tris, pH 6.8, 100% glycerol, and 0.05% bromophenol blue. The samples were loaded into wells of 8% SDS-polyacrylamide containing 1% gelatin. Electrophoresis was performed in a Bio-Rad apparatus at 80 V for 2 h, until the bromophenol blue reached the bottom of the gel. The gel was then incubated at 37°C overnight in activation solution consisting of 50 mM Tris, pH 8.4, 5 mM CaCl_2_ and ZnCl_2_. Staining with 0.5% Coomassie blue, 30% MeOH, and 10% acetic acid was performed for 2 h until clear bands over a dark background were observed. The gels were photographed, and the intensity of gelatinolytic action (clear bands) was analyzed in a UVP, UV, White Darkhon image analyzer.

Evaluation of cytokine production and adhesion molecules: The production of tumor necrosis factor alpha (TNF-α), interferon gamma (IFN-γ) and interleukin 10 (IL-10) were evaluated. Briefly, 60 mg of cardiac tissue sample was homogenized and solubilized in a solution containing 50 mM potassium phosphate buffer, pH 7.4, 0.3 M sucrose, 0.5 mM DTT, 1 mM EDTA, pH 8.0, 0.3 mM PMSF, 10 mM NaF, and 1∶100 protease inhibitor. Cytokine levels in cardiac homogenate were evaluated by ELISA, according to the manufacturer’s instructions (R & D Systems, Minneapolis, MN, USA) [17].

Cardiac lipid hydroperoxide and antioxidant enzyme analysis: lipid hydroperoxide (LH) based on the hydro peroxide-mediated oxidation of Fe^2+^; and antioxidant enzyme activities [23]. Glutathione peroxidase (GSHPx, E.C.1.11.1.9), superoxide dismutase (SOD, E.C.1.15.1.1) and catalase (CAT, E.C.1.11.1.6) activities were assessed as previously described [23], [24], [25]. Enzyme activity assays were performed at 25°C using a micro-plate reader (lQuant-MQX 200 with Kcjunior software connected to computer system control, Bio-Tec Instruments, Winooski, Vermont, USA). The absorbance was measured using a Pharmacia Biotech spectrophotometer (UV/visible Ultrospec 5000 with Swift II Applications software connected to computer system control, 974213, Cambridge, England, UK) at 560 nm. All reagents were purchased from Sigma (St. Louis, Missouri, USA) [23].

Statistical analysis: Comparisons between groups were made using Student’s t test for parameters with normal distribution. Otherwise, groups were compared using the Mann-Whitney U test. Data were expressed as means ± SD or medians (including the lower quartile and upper quartile). Data analysis was carried out using SigmaStat for Windows v2.03 (SPSS Inc, Chicago, IL). The significance level was 5%. This study was designed to have 80% power of detecting a difference of several parameters between the mean for the control animals and the mean for taurine-deficient animals: left ventricular end-diastolic diameter adjusted by body weight (2 mm/kg); and cross sectional area (30 µm^2^), left ventricular systolic diameter adjusted by body weight (0.2 mm/kg).

## Results

The groups did not differ with regard to final body weight (C = 257 (254–277) g, T(-) = 262 (252–288) g; p = 0.558). Group T(-) animals had lower levels of taurine in the left ventricle (C = 1.8±0.8, T(-) = 0.4±0.1 µmol/mg of tissue; p = 0.007).

The morphological data assessed by echocardiogram are shown in Table 1. Taurine deficiency resulted in lower values of LV posterior wall thickness and the ratio of LV wall thickness (LVWT)/LV end-diastolic diameter (LVDD). There were no differences in the other morphological echocardiographic variables between the groups.

**Figure 1:**
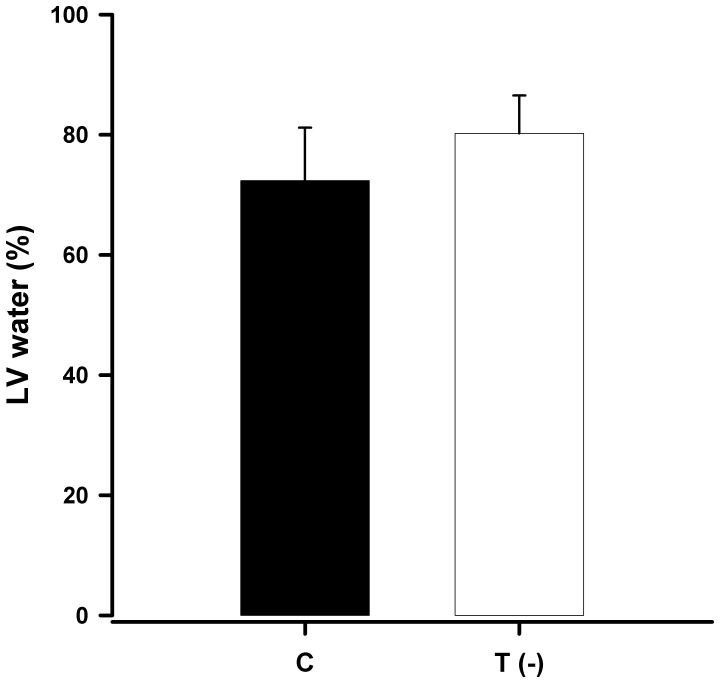
Bar graph representing the mean and standard deviation of water content (%) in the left ventricles of control and taurine-deficient animals (P = 0.03).

The functional data assessed by echocardiogram are shown in Table 2. LV systolic diameter, heart rate, ejection fraction and fractional shortening were decreased in the T(-) group, compared to the C group. In contrast, the T(-) group had increased E/A ratios.

Histological analysis data and metalloproteinases values are shown in Table 3. The CSA was decreased in the T(-) group compared to the C group. There were no differences in the collagen volume fraction or metalloproteinase values between the groups.

**Figure 2:**
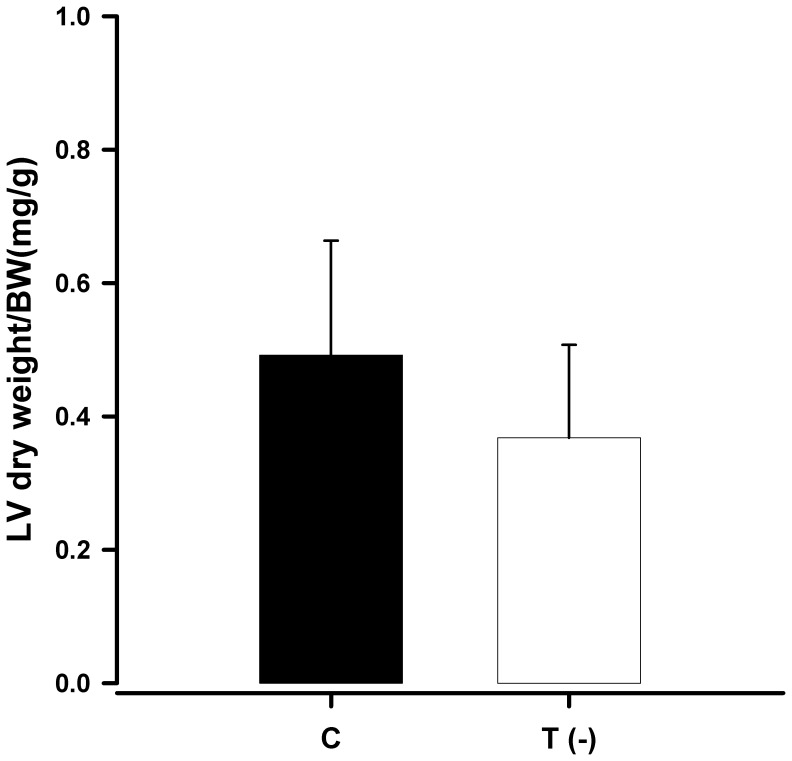
Bar graph representing the mean and standard deviation of the left ventricular (LV) dry weight and normalized to body weight (BW) of control animals and taurine-deficient animals (P = 0.05).

The LV water content was higher (Figure 1) and the LV dry weight was lower in the T(-) group compared to the C group (Figure 2).

**Table 3:** Histopathology by light microscopy, myocyte cross-sectional area, collagen value fraction and determination of metalloproteinases -2 and -9 activation.

	Group Cn = 7	Group T(-)n = 7	P
**CSA (µm^2^)**	238.3±24.2	196.3±29.7*	**0.013**
**CVF (%)**	1.37±0.8	1.34±1.3	0.955
**MMP2 A/I**	0.51±0.1	0.51±0.1	0.870
**MMP9 A/I**	0.65±0.7	0.60±0.4	0.898

Group C: control animals, Group T (-): taurine-deficient animals; CSA: myocyte cross sectional area; CVF: collagen value fraction; MPP2 A/I: ratio of the active and inactive forms of metalloproteinase 2; MPP9 A/I: ratio of the active and inactive forms of metalloproteinase 9. Data are expressed as means ± standard deviations or medians (quartile 1 - quartile 3). Bold values indicate statistical significance (P≤0.05).

The data showing the cardiac lipid hydro peroxide and antioxidant enzyme activities are presented in Table 4. The T(-) group showed a higher concentration of lipid hydro peroxide and a lower catalase and glutathione peroxide activity than the C group. No differences were observed for cytokine and adhesion molecule levels (Table 5).

**Table 4:** Oxidative stress evaluation.

	Group Cn = 7	Group T(-)n = 7	P
**LH (nmol/g of tissue)**	158.16±14.36	197.75 33.29	**0.023**
**GPx (µmol/g of tissue)**	55.95±7.83	35.73±7.42	**<0.001**
**SOD (nmol/mg of protein)**	20.03±2.41	20.57±1.80	0.673
**Catalase (µmol/g of tissue)**	112.58±24.64	77.40±16.42	**0.016**

Group C: control animals, Group T (-): taurine-deficient animals; LH: Lipid hydro peroxide; GPx: glutathione peroxidase; SOD: superoxide dismutase. Data are expressed as means ± standard deviations. Bold values indicate statistical significance (P≤0.05).

**Table 5:** Cytokine production and adhesion molecules.

	Group Cn = 7	Group T(-)n = 7	P
**TNF-α (pgm/mg of protein)**	21.7 (19.2–29.2)	21.7 (16.6–28.4)	0.613
**INF-γ (pgm/mg of protein)**	9.38 (5.33–12.68)	9.44 (7.85–12.23)	0.867
**Il-10 (pgm/mg of protein)**	33.5±8.4	34.4±16.6	0.894
**ICAM-1 (pgm/mg of protein)**	109.6±41.9	85.9±35.0	0.254

Group C: control animals, Group T (-): taurine-deficient animals, TNF-α: tumor necrosis factor alpha, INF-γ: interferon-gamma, Il-10: interleukin 10; ICAM-1: intercellular adhesion molecule 1. Data are expressed as means ± standard deviations or medians (quartile 1 - quartile 3).

## Discussion

The rat taurine deficiency model used in this study consisted of treatment with a solution of 3% ß-alanine. The treatment caused a 77% decrease in the concentration of taurine in the LV. This decrease in taurine concentration is higher than those observed by Parildar et al. [14] and Dawson Jr. et al. [15] (20% and 50% respectively). The difference can be explained by the age of the animals used in the experimental protocols. Parildar et al. [14] used rats at 22 months, and Dawson, Jr. et al. [15] used rats weighing 180 to 200 g. The animals studied by Dawson Jr. et al. [15] were 2 to 3 weeks older than the rats used in the present study. In a review article, Schuller-Lewis & Park [26] demonstrated that young animals show a lower capacity for taurine synthesis when compared to older animals. Thus, the observed differences between studies may be due to animal age differences. In humans, ß-alanine is used as an ergogenic supplement. The principal role is acting as a substrate of carnosine synthesis in skeletal muscle (a major contributor to H^+^ buffering during high-intensity exercise) [27]. ß-alanine supplementation-induce increase in muscle carnosine and the result is the opposite effect of the muscle taurine depletion [28]. It is not known if ß-alanine/carnosine has compensatory changes in the heart. However, animals treated with ß-alanine and decreased taurine levels shows a lipid peroxidation potential in the heart [14].

The noteworthy finding in the present study was that taurine deficiency resulted in cardiac atrophy, as confirmed by thinning of the ventricular wall, reduced left ventricular dry weight, decreased myocyte cross sectional area, and increased oxidative stress. Regarding diastolic function, these data are consistent with decreased diastolic function in animals that are deficient in taurine. Indeed, the taurine-deficient group showed a lower velocity A and E, a higher E/A ratio, and a tendency for lower IRT/HR. With regard to systolic function, the echocardiographic data are consistent with systolic dysfunction. Decreased ejection fraction, fractional shortening and cardiac output were observed in the taurine-deficient group, compared to the control group. Moreover, in the present study, increased fibrosis and greater activation of metalloproteinases were not observed upon optical microscopy. These findings are similar to those described by Ito et al. [29] in taut−/− transgenic mice. The mice displayed small areas of atrophied cardiac myocytes, an absence of fibrosis, eccentric remodeling and systolic dysfunction [29].

Baskin & Taegtmeyer (2011) stated that these atrophic remodeling characteristics are cellular consequences of metabolic and hemodynamic unloading of a stressed heart [30]. Therefore, the atrophic cardiac remodeling observed in our study may result from two possible sources: 1) metabolic unloading (food restriction, protein-energy malnutrition) [31], abnormal protein metabolism [32] and inflammation [33] or 2) hemodynamic unloading [34], .

Food restriction and protein-energy malnutrition induce changes in the heart such as atrophy and cardiac dysfunction [36]. Animal studies with food restriction and cachectic rats showed lower body weight, lower LV weight, a higher collagen concentration, and no change in cardiac performance [31], [37]. Other common findings in malnourished animals include bradycardia, hypotension and reduced cardiac output [38]. Thus, our data are similar to those found in malnourished rats. However, our animals suffered no food restrictions, and no differences in body weight or collagen percentage were observed.

Another mechanism that could explain our study findings is a decrease in protein synthesis and/or increase in protein catabolism. Protein synthesis is dependent on amino acids, and decreased protein synthesis may occur when amino acids are limiting, such as in the case of deficient diet or amino acid overuse (catabolic stress) [32]. For example, sulfur-containing amino acids such as methionine and cysteine can be limiting. However, this is not observed in taurine deficiency. Taurine is not incorporated into proteins; rather, it is the end product of the metabolic pathway of methionine [32]. Another possible mechanism of taurine is to regulate intracellular signaling pathways that are involved in protein synthesis. An example of this mechanism is the action of inhibitors of the renin angiotensin converting enzyme in the protein synthesis and catabolism promoting a reduction of left ventricular mass [39]. However, unlike other sulfur-containing amino acids, taurine does not participate in signaling pathways that control protein turnover [32].

Thus, our results suggest that the mechanisms that led to cardiac atrophy and ventricular dysfunction in taurine-deficient animals are not the same mechanisms involved in food restriction, protein-energy malnutrition or protein metabolism alteration.

Another potential mechanism involved in cardiac atrophy is load alterations. Indeed, changes in the hemodynamic mechanism of myocardium "load" may result in structural remodeling. In situations in which the heart undergoes pressure overload, concentric hypertrophy is observed, whereas volume overload leads to eccentric hypertrophy [34]. In the latter situation, dilatation of the cavity, changes in geometry, ventricular dysfunction and increased cardiac mass can be observed [34]. In contrast, atrophy occurs in situations in which there is a reduction in afterload [34]. Left ventricular mass regression is also observed when blood pressure is reduced [40]. However, in this study, no blood pressure measurements were made, although a worsening of systolic and diastolic function was observed. In studies in which animals underwent the same model of taurine deficiency, no differences in blood pressure were observed when compared with non-deficient animals [41], [42]. Thus, it is unlikely that the changes observed in this study are due to a reduction in blood pressure.

Inflammation was not responsible to the cardiac dysfunction observed in our study. Since the findings atrophy with decreased cardiac myocyte area, absence of fibrosis, no differences in metalloproteinase activity and no cytokine profile differences are not compatible with changes due to inflammation.

So, considering the critical role of oxidative stress in cardiac remodeling, we suggest that oxidative stress is associated with the cardiac dysfunction observed in our study. Oxidative stress has direct effects on cellular structure and function, and it can activate signaling molecules that are involved in cardiac remodeling, including apoptotic cascade [43], [44]. In previous studies, taurine has been shown to promote antioxidant activity, regulating the rate of ROS generation by the mitochondria [8]. Gokce et al. reported that taurine significantly inhibited glutathione depletion and DNA damage caused by buthionine sulfoximine, an effective GSH-depleting compound [45]. Additionally, Ghosh et al. showed that taurine prevents arsenic-induced cardiac oxidative stress in cardiomyocytes [46]. Thus, the heart could be more susceptible to oxidative stress in taurine-deficient animals [14]. However, although the association between oxidative stress and remodeling, this study provides no evidence that there is causal relationship between oxidative stress and cardiac function and further study is needed to test this hypothesis.

In conclusion, taurine deficiency promoted structural and functional cardiac alterations, particularly with regard to the left ventricle.
